# A systematic review with meta-analysis of the effects of smoking cessation strategies in patients with rheumatoid arthritis

**DOI:** 10.1371/journal.pone.0279065

**Published:** 2022-12-15

**Authors:** Maria A. Lopez-Olivo, Gaurav Sharma, Gagandeep Singh, Justin James, Kate J. Krause, Paul Cinciripini, Robert J. Volk, Maria E. Suarez-Almazor

**Affiliations:** 1 Department of Health Services Research, The University of Texas MD Anderson Cancer Center, Houston, Texas, United States of America; 2 Department of Internal Medicine, University of South Alabama, Mobile, Alabama, United States of America; 3 Government Medical College, Amritsar, India; 4 City University of New York School of Medicine, New York, New York, United States of America; 5 Research Medical Library, The University of Texas MD Anderson Cancer Center, Houston, Texas, United States of America; 6 Department of Behavioral Science, The University of Texas MD Anderson Cancer Center, Houston, Texas, United States of America; 7 Department of General Internal Medicine, The University of Texas MD Anderson Cancer Center, Houston, Texas, United States of America; University of Wisconsin-Madison, UNITED STATES

## Abstract

**Objective:**

Smoking rates among patients with rheumatoid arthritis (RA) exceed those in the general population. This study identified smoking cessation strategies used in patients with RA and synthesized data on their effects.

**Methods:**

We conducted a systematic review of studies that reported effects of interventions for smoking cessation in patients with RA. We searched 5 electronic databases until March 2022. Screening, quality appraisal, and data collection were done independently by 2 reviewers.

**Results:**

We included 18 studies reporting interventions for patients or providers: 14 evaluated strategies for patients (5 education on cardiovascular risk factors including smoking, 3 educational interventions on smoking cessation alone, 3 education with nicotine replacement and counseling, and 1 study each: education with nicotine replacement, counseling sessions alone, and a social marketing campaign). Smoking cessation rates ranged from 4% (95% CI: 2%-6%, 24 to 48 weeks) for cardiovascular risk education to 43% (95% CI: 21%-67%, 104 weeks) for counseling sessions alone. The pooled cessation rate for all interventions was 22% (95% CI: 8%-41%, 4 weeks to 104 weeks; 9 studies). Four interventions trained providers to ascertain smoking status and provide referrals for smoking cessation. The pooled rates of referrals to quit services increased from 5% in pre-implementation populations to 70% in post-implementation populations.

**Conclusion:**

Studies varied in patient characteristics, the interventions used, and their implementation structure. Only 3 studies were controlled clinical trials. Additional controlled studies are needed to determine best practices for smoking cessation for patients with RA.

## Introduction

Smoking is the leading cause of preventable disease. More than 8 million people a year die from diseases related to tobacco use [1]. In 2020, 22% of the world population (approximately 1.3 billion) were current tobacco users [2]. Smoking thus poses a great global economic burden, leading to an annual net loss equivalent to 1.8% of the gross domestic product in the world, owing in large part to lost productivity and increased use of health services [3].

Rheumatoid arthritis (RA) is a chronic condition that typically starts in middle-age and is slightly more frequent in women. The disease has a major impact on quality of life. It is associated with significant comorbidities and early mortality. No curative therapy is available, and in most cases, treatment is required for life. It affects approximately 1% of the global population and poses a great economic burden to society [4,5].

Smoking rates among patients with rheumatoid arthritis (RA) have been reported to be as high as 30%, exceeding the overall smoking rates reported in general populations [6,7]. Previous epidemiological studies have identified smoking as an important risk factor for RA [8–10]. The risk has been estimated 40% higher among ever smokers compared to never smokers [8]. In 2001, a study reported more than 65% of people living with RA have smoked cigarettes [11]. By 2016, this number had increased to more than 80% [12]. Furthermore, evidence suggest that half of the patients with RA are current smokers at the time of disease onset [11] and up to 26% continue to smoke despite the risk for increased RA complications, hospitalizations due to cardiovascular disease and respiratory tract infections, osteoporosis, and poor response to treatment [13,14]. Factors associated with smoking persistence in patients with RA are thought to be a combination of biological, psychological and social factors including managing RA pain, using smoking as a coping strategy, behavior carried from adolescence, and/or limited knowledge about the effects of smoking on the disease [15].

Current guidelines on smoking cessation recommend clinicians to ask about tobacco use, advise them to stop using tobacco, and provide behavioral intervention (i.e., counseling) in addition to approved pharmacotherapy for cessation [16]. For patients with RA, the benefits of smoking cessation are numerous [17] including slowing down the progression of chronic obstructive pulmonary disease [18], reduced risk of fractures after 10 years of quitting [19], risk of dying from lung cancer halved within ten years [20], the risk of hospitalization for cardiovascular events or respiratory tract infections decreasing for each additional year of smoking cessation [14], and reduced mortality risk within four years [21]. Immediate health benefits have also been reported such as improved blood pressure and lung function [22,23], improved disease activity, better response to RA treatment, reduced infective risks of immunosuppressive therapy, and improved success of medication dose reduction [17]. However, despite current guidelines on smoking cessation, only about 10% of rheumatology visits with patients who smoke include documentation of cessation counseling or follow-up advice [24]. The purpose of this systematic review of the literature was to identify smoking cessation strategies used in patients with RA and to synthesize data on smoking cessation outcomes, referrals to quit services, knowledge about cessation benefits, and health outcomes.

## Methods

### Registration

This systematic review was conducted following Cochrane methodological standards [25], and results were reported according to the Preferred Reporting Items for Systematic Reviews and Meta-Analysis (PRISMA) statement [26]. The protocol of this systematic review was submitted to the International Prospective Register of Systematic Reviews (PROSPERO) under registration number CRD42020215287 (https://www.crd.york.ac.uk/prospero/display_record.php?RecordID=215287).

### Eligibility criteria

We included experimental (i.e., controlled trials [randomized or not], uncontrolled studies [i.e., same population before and after], and implementation studies [i.e., different population before and after]) that reported the effects of smoking cessation interventions in adult patients with RA in any setting and were published in abstract or full-text format. We included any smoking cessation strategy reported by the authors. We excluded basic science studies (i.e., in vivo, in vitro, or animal studies), studies with different population (i.e., juvenile idiopathic arthritis or not RA), reporting development of methods, opinion pieces (i.e., not original research), or reviews. We also excluded studies not reporting data on the effects of the smoking cessation strategy being evaluated (i.e., studies not directly reporting quantitative data on the intervention). Therefore, qualitative studies, articles reporting tool development without evaluation, case reports, guidelines, protocols, and surveys reporting the prevalence of tobacco use in RA were excluded.

### Information sources

We searched the literature in 5 electronic databases (MEDLINE, EMBASE, Cochrane, CINAHL, and Web of Science) for articles published from inception until March 2022. Sources of gray literature (unpublished records) were searched through ClinicalTrials.gov. In addition, the reference lists of included articles were hand-searched to identify other potentially relevant studies.

### Search

We used a broad search strategy to capture all available studies, including terms related to smoking cessation and RA. An expert librarian (KJK) built the search strategy with input from the study team. Our search was limited to humans, but no other restrictions were imposed. S1 Table shows our search strategy for MEDLINE.

### Study selection

For study selection, we followed a 2-step process using DistillerSR (Evidence Partners, Ottawa, Canada; https://www.evidencepartners.com). During the first step, two authors (GS and GS) independently screened titles and abstracts for possible inclusion. For the second step, relevant citations were retrieved in full text to determine the final eligibility for inclusion. Reasons for exclusion were independently recorded, and disagreements were resolved through discussion or, when needed, by a third author (MLO).

### Data collection process

Two authors independently collected the data (GS, GS). Disagreements were resolved by consensus or, when needed, consultation of a third author (MLO). If more than 1 publication reported on the same study, the most recently published results were used. We contacted study authors when clarification was needed regarding any missing outcome data or inconsistencies. Data collected for analyses has been shared in the Open Science Framework repository (https://osf.io/hruyz/?view_only=887674593c43432aac52ddd74b93440b).

### Data items

Data collected included: (i) study characteristics (author, year of publication, country, number of centers, funding, methods to assess risk of bias, design), (ii) participants’ characteristics (age, gender, ethnicity, socioeconomic status, pack-years, eligibility criteria), (iii) intervention characteristics (description, provider, timing, duration, etc.), and (iv) outcome data (number of events and number of participants per treatment group for dichotomous outcomes; mean and standard deviation and number of participants per treatment group for continuous outcomes). Our primary outcome was the smoking cessation rates as reported by the authors. Other outcomes included smoking cessation-relate outcomes (i.e., percent abstinent, reduction in the number of cigarettes, referrals to cessation services, assessment of smoking status by providers, number of cigarettes smoked), knowledge about cessation benefits, and RA-related outcomes (i.e., disease activity measured by the Disease Activity Score [27], pain [28], global health assessment [29,30], and function measured by the Health Assessment Questionnaire [31]).

### Risk of bias in individual studies

Two authors independently appraised the quality of the studies (GS, GS). Disagreements were resolved by consensus or, when needed, consultation of a third author (MLO). The risk of bias tool (RoB 2.0) for randomized controlled trials and the risk of bias in nonrandomized studies (ROBINS-I) were used to appraise study quality, and the RoBvis tool was used to generate traffic-light plots [32]. The RoB 2.0 questions evaluate the randomization process, the effect of assignment and adherence to the intervention, missing outcome data, measurement of outcomes, and selection of the reported result. Each item was judged as having a high or low risk of bias, some concerns, or no information. ROBINS-I was used to assess bias caused by confounding, selection of participants, classification of the interventions, deviations from intended interventions, missing data, measurements of outcomes, and selection of reported results. These domains were judged as having a low, moderate, serious, or critical risk of bias, or no information. An overall judgment of the risk of bias was obtained using domain-level judgments. A study with a critical risk of bias in any single domain or a serious risk of bias in 2 or more domains was judged to have an overall critical risk of bias.

### Summary measures

Dichotomous data were analyzed as risk ratios with their corresponding 95% confidence intervals (CIs). Continuous data were analyzed as mean differences (MD) with their corresponding 95% CI. For studies without a comparison group, rates were pooled and their 95% CI was calculated. Similarly, for implementation studies with different participants assessed before and after intervention, we pooled the rates (before and after the implementation) separately without a test of difference and calculated their 95% CI.

### Synthesis of results

#### Eligibility for synthesis

Data were pooled if at least 2 studies reported on the same outcome regardless of when was the follow-up assessed. Combining different follow-up times allowed us to determine an estimate independent of time. However, we also evaluated differences between short and long-term studies (≤6 vs > 6 months).

#### Preparing for synthesis

A random-effects model was used to pool studies. To pool rates of uncontrolled studies, we used the Freeman-Tukey arcsine transformation to stabilize variances and conduct a meta-analysis using inverse variance weights. The resulting estimates and CI boundaries were back-transformed into proportions. Separate analyses were performed for before-and-after studies using the Cochrane methodology to pool paired MDs. An imputed conservative correlation coefficient of 0.8 was used when the within-groups correlation coefficient was not reported [33]. When studies did not report means, we used the median values [34]. Ranges were transformed into standard deviations (SDs) using previously validated methods [35]. Mean and SD were calculated from a frequency distribution with intervals using the formulas: mean = SUM(FREQ*interval midpoint)/SUM(FREQ) and SD = SQRT((n*SUM(FREQ*mean^2^)—SUM(FREQ*mean)^2^)/n(n-1).

#### Statistical and synthesis methods

All statistical tests performed were 2-sided, and we considered a *P* value of less than 0.05 statistically significant. Analyses were conducted using STATA version 15 (StataCorp LP, College Station, TX).

#### Methods to explore heterogeneity

Study heterogeneity was assessed by using the I^2^ statistic. An I^2^ value greater than 50% was considered to indicate substantial inconsistency. Subgroup analyses were performed to investigate whether study characteristics (design, follow-up, and type of intervention and control used) could explain the inconsistency observed.

#### Sensitivity analysis

Sensitivity analyses were performed to assess the robustness of the methods used to impute measures of dispersion (i.e., SD or 95% CI) and the impact of excluding studies with a high risk of bias.

### Risk of bias across studies

The risk of publication bias was assessed through funnel plots and an Egger regression test when 10 or more studies reporting on the same outcome were available.

### Certainty assessment

A summary-of-findings table was created following the GRADE approach to rate the quality of the evidence for each outcome [36]. We expressed certainty using 4 categories: (i) high quality of evidence, that is, further research is very unlikely to change our confidence in the effect estimate; (ii) moderate quality, that is, further research is likely to have an important impact on our confidence in the effect estimate and may change the estimate; (iii) low quality, that is, further research is very likely to have an important impact on our confidence in the effect estimate and is likely to change the estimate; and (iv) very low quality, that is, we are uncertain about the estimate.

## Results

### Study selection

#### Flow of studies

**Fig 1:**
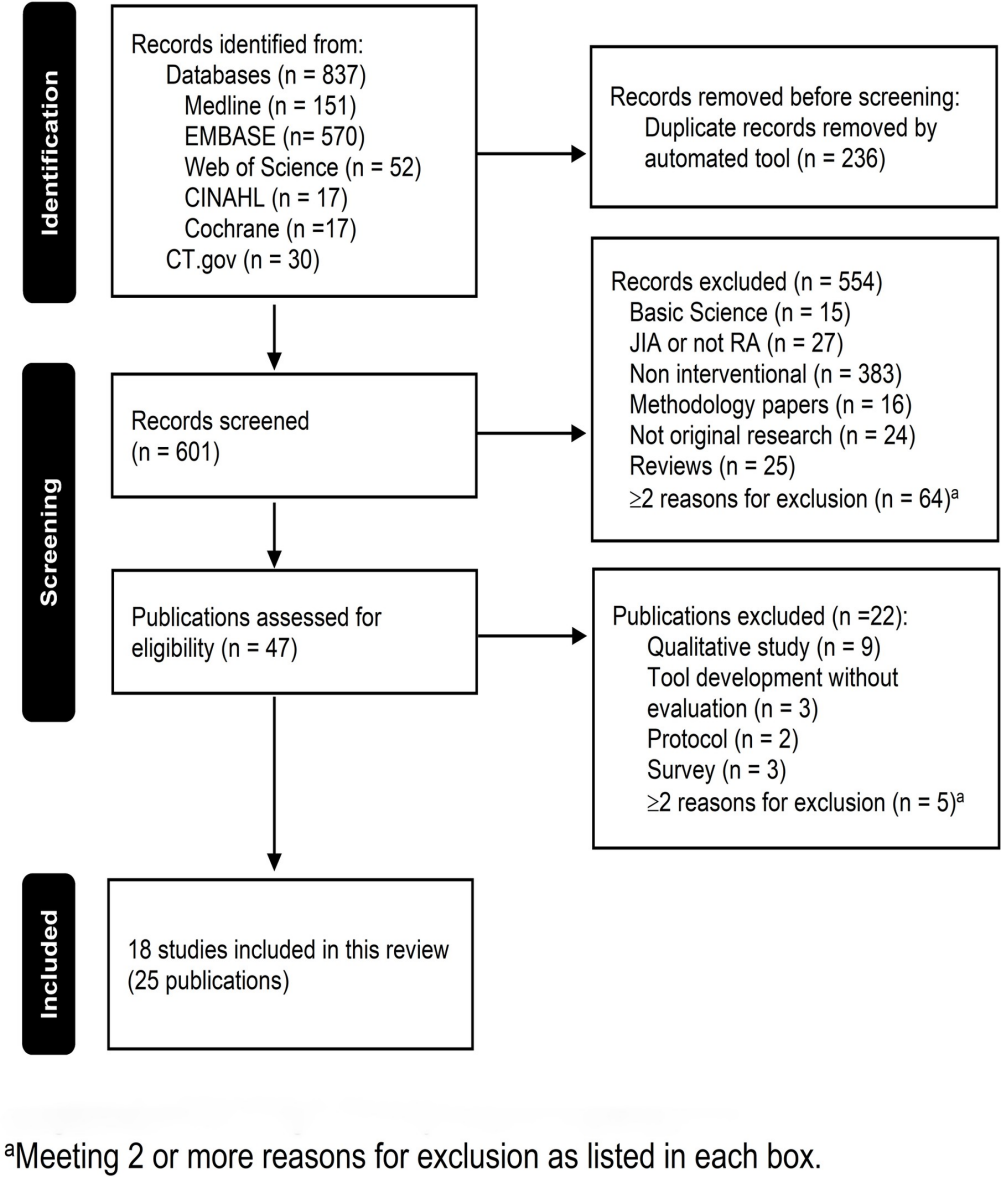
Diagram of study selection. CT.gov, ClinicalTrials.gov; JIA, juvenile idiopathic arthritis.

We retrieved 837 citations. After removal of duplicates, 601 abstracts were screened. Of these, only 47 moved to the full-text screening. Fig 1 shows the diagram of study selection with the reasons for exclusion in each phase. Twenty-five publications (18 studies) met our study eligibility criteria [37–61].

#### Excluded studies

The list of excluded studies is shown in S2 Table.

### Study characteristics

Among the 18 studies included in the review, 10 studies were reported in abstract format (Table 1). Three studies were randomized controlled trials [37,46,58], and the rest were nonrandomized studies: 10 were uncontrolled trials (same participants evaluated at baseline and follow-ups) [42,44,47,48,50–54,59], and 5 were implementation studies (different participants evaluated before and after implementation of the intervention) [38–41,45]. Sample sizes ranged from 20 to 970. Seven studies included patients with RA and other conditions [38–40,50,51,54,59].

**Table 1:** Study characteristics.

Study	Country	No. of centers	Sample size, No.^a^	Population	Follow-up	Recruitment Period	Outcome(s) reported^e^	Funding
***Randomized controlled trials***								
**Aimer 2017 [37,57]**	New Zealand	1	38 (I = 19, C = 19)	All RA	6 months	2012–2014	Smoking cessation Reduction in cigarette consumption	New Zealand Health Research Council and Arthritis New Zealand. University of Otago Research Fund
**John 2013 [46]**	UK	1	110 (I = 52, C = 58)	All RA	6 months	2010	Current smoking Knowledge	Arthritis Research UK
**Soubrier 2013 [56,58]** ^**b**^	France	20	970 (I = 488, C = 482)	All RA (ACR 1987 criteria)	6 months	2011	Smoking cessation	French National Research Program. Roche Ltd France
***Uncontrolled trials (same participants assessed at baseline and follow-up)***								
**Al Hamarneh 2021 [59]**	Canada	17	99	Rheumatic diseases (55 with RA)	6 months	2017–2019	Smoking status	Canadian Initiative for Outcomes in Rheumatology
**Gordon 2001, 2002 [42,43]**	UK	1	22	RA, starting treatment with sulfasalazine	48 weeks	NR	Smoking cessation Reduction in cigarette consumption	NR
**Gudelj Gracanin 2014 [44]** ^**b**^	Croatia	2	96	RA (1987 criteria)	1 month	NR	Smoking cessation Pain, DAS28-CRP	None
**Karlsson 2014 [47]** ^**b**^	Sweden	1	40	Early RA or starting a first biological treatment	2 years	2011–2013	Smoking cessation Reduction in cigarette consumption Smoking status Pain, global health, HAQ	NR
**Khan 2017 [48]** ^**b**^	Ireland	1	180	RA (1987 criteria)	6 months	2016–2017	Smoking cessation DAS28-CRP	NR
**Naranjo 2013, 2014 [49,50]**	Spain	1	152	Inflammatory rheumatic diseases (55 with RA)	12 months	2011	Smoking cessation^c^ Reduction in cigarette consumption^c^ Smoking status	None
**Sadhana Singh Baghel 2016, 2017 [51,55]** ^**b**^	India	1	211	Rheumatology patients seen in outpatient department (162 with RA)	NR	NR	Smoking cessation	None
**Tekkatte 2016 [52]** ^**b**^	UK	1	20	Established RA	NR	NR	Knowledge	None
**Thomas 2015 [53]** ^**b**^	UK	1	100	NR	1 year	2011–2012	Smoking status	None
**Zeun 2015 [54]** ^**b**^	UK	1	202	Rheumatology patients (62 with RA)	1 year	NR	Smoking cessation^d^	None
***Implementation studies (different participants assessed before and after intervention)***								
**Bartels 2017 [38,60]**	USA, Wisconsin	3	B = 135, A = 421	Rheumatology patients seen in 3 clinics (unspecified # for RA)	6 months	2012–2016	Referral to the Quit line	None
**Brandt 2020 [39,61]** ^**b**^	USA, Atlanta	1	B = 535, A = 123	Rheumatology patients with tobacco use (unspecified # for RA)	3 months	NR	Referral to the Quit line Smoking status	NR
**Chodara 2018 [40]** ^**b**^	USA, Wisconsin	1	B = 100, A = 129	Rheumatology patients seen in 1 clinic (unspecified # for RA)	NR	2015–2018	Referral to the Quit line	NR
**Chow 2019 [41]**	New Zealand	1	B = 53, A = 100	All RA	NR	2004–2016	Referral for smoking cessation Reduction in cigarette consumption Smoking status	NR
**Harris 2016 [45]**	UK, Scotland	1	B = 306, A = 340	Seropositive RA attending UK National Health Service	NR	NR	Reduction in cigarette consumption Smoking status Knowledge	Pfizer

DAS28-CRP, Disease Activity Scale 28 Joints–C Reactive Protein; HAQ, Health Assessment Questionnaire–Disability Index; NR, not reported; RA, rheumatoid arthritis.

^a^Numbers reflect the total number of patients included in the study (i.e., people who smoke [active, passive, current, past] or people who do NOT smoke), independently if they received the intervention or not.

^b^Publication was a conference abstract.

^c^Total abstinence in the last 7 days; Reduction in cigarette consumption by ≥30% or at least 50%.

^d^Total abstinence for at least 2 weeks, verified by a carbon monoxide reading of 0–6 parts per million.

^e^Outcomes reported relevant to this review.

### Participants’ characteristics

Mean age ranged from 50 to 65 years (S3 Table). The percentage of female participants ranged from 51% to 91%. In 7 studies, all participants were current tobacco users [37–40,47,48,50]. The remaining studies included a mixture of former and current tobacco users, with rates of current tobacco users ranging from 11% to 36%.

### Intervention characteristics

The studies were divided by type of intervention (Table 2). There were 6 types of interventions targeting patients: 1 study reporting on a social marketing campaign [45]; 3 reporting on a health education intervention alone [44,51,52], 1 reporting on health education combined with nicotine replacement therapy (NRT) [48], 3 reporting on health education combined with NRT plus counseling sessions [37,50,54], 1 reporting on counseling sessions alone [47], and 4 reporting on lifestyle education focused on cardiovascular risk with goal setting [43,46,53,56,59]. There were 2 types of interventions targeting providers: 3 studies (18%) reported using electronic health record prompts to assess smoking status and offer training on how to refer to Quit line support services [38–40], and 1 study reported using a screening questionnaire to assess smoking status to offer referrals [41].

**Table 2:** Intervention characteristics.

Study	Description of intervention	Provided by	Duration	Follow-up assessment	Comparison groups or subgroup analysis
**Social marketing campaign**					
**Harris 2016 [45]**	Postcards mailed, poster in outpatient departments, press statement, website, newspaper and radio	Not applicable	Not specified	3–12 months following the campaign	-
**Health Education**					
**Gudelj Gracanin 2014 [44]**	Short, spoken advice about the harms of smoking and advice to quit smoking	Not specified	Once	1 month	-
**Sadhana Singh Baghel 2014, 2016 [51,55]**	Information about methods of quitting tobacco and its importance	Not specified	Once	Not specified	Passive vs active tobacco users
**Tekkatte 2016 [52]**	Information provided about the increased risk of RA, severe disease, and poor response with smoking	Not specified	Once	Immediately after	-
**Health education + NRT**					
**Khan 2017 [48]**	Face-to-face advice, handout, and NRT	Not specified	Once	6 months	Gender, age, BMI, disease activity
**Health education + NRT + Counseling sessions**					
**Aimer 2017 [37]**	Face-to-face educational session, educational handout explaining the effect of smoking on RA	ABC^a^ by the rheumatology clinical nurse specialist. Additional education by community-based arthritis educators trained in smoking cessation	3 follow-up telephone calls, a support website, and 12 weekly smoking cessation advice e-mails with education^b^	6 months	Health education + NRT + Counseling sessions vs Health education + NRT
**Naranjo 2013, 2014 [49,50]**	Verbal or written advice on the benefits of quitting smoking + written documentation with helpful tips on how to quit smoking + NRT if needed	Rheumatologist	Telephone follow-up visit in the 3rd month by the rheumatology nurse	12 months	Active vs former tobacco users
**Zeun 2015 [54]**	An information booklet in waiting area^c^ + behavioral support sessions	Smoking advisor at the point of rheumatology contact	Patients attended counseling for 6 weeks^d^	12 months	Patients attending the rheumatology service versus those attending the service of their preference
**Counseling sessions**					
**Karlsson 2014 [47]**	Individualized smoking cessation support	Rheumatology nurse with special training in motivational interviewing and smoking cessation	Contact every 4 weeks	2 years	Patients who continue to tobacco users vs those quitting
**Lifestyle education (in the context of CVD risk) + goal setting**					
**Al Hamarneh 2021 [59]**	Individualized CVD risk assessment and education	Pharmacist	Monthly follow-up to check on progress and provide ongoing care and motivation	6 months	-
**Gordon 2001, 2002 [42,43]**	Advised on CVD risk including generic quitter’s pack (further advice and telephone numbers)	General practitioner	Clinic appointments dealing with lifestyle factors every 12 weeks	48 weeks	-
**John 2013 [46]**	Small-group education on CVD risk	Rheumatology research registrar with interest in patient education	8-week course^e^	6 months	Participants vs waiting list candidates^f^
**Soubrier 2013 [56]**	Advised on CVD risk management + report^g^ sent to physician and rheumatologist	Nurse^h^	Not specified	6 months	Nurse-led program vs patients receiving a video on joint self-assessment
**Thomas 2015 [53]**	Lifestyle assessment + CVD risk + health promotion advice and literature	Not specified	Patients with high risk of CVD were offered 6 sessions to support goals (including smoking cessation)	12 months	-
**Interventions targeting providers**					
**Study**	**Intervention**	**Target population**	**Description**	**Implementation period**	**Objective**
**Bartels 2017 [38]**	Quit Connect protocol training	Nurses and medical assistants	See footnote^i^	6 months	To measure process steps
**Brandt 2020 [39]**	Quit Connect protocol training	Staff	See footnote^i^	3 months	To determine performance and rates of triage
**Chodara 2018 [40]**	Quit Connect protocol training (1-h session + monthly feedback on fidelity)	Medical assistants	See footnote^i^	3 years	To determine delivery of protocol components per patient visit
**Chow 2019 [41]**	Brief screening questionnaire completed in the waiting room prior to the clinic appointment	Reception staff	People who smoke were offered referral for smoking cessation support available free of charge	6 years	To assess smoking status and offer referrals

BMI, body mass index; CVD, cardiovascular disease; EHR,electronic health record system; NRT, nicotine replacement therapy.

^a^ABC (Ask, Brief advice, Cessation support) = brief advice and subsidized NRT for 8 weeks.

^b^Education about smoking and RA, pain control, exercise, coping, and support.

^c^About the impact of smoking in various rheumatological conditions.

^d^During the first session the counselor asked about smoking behavior and previous attempts to quit, assessed nicotine dependence and discussed stop-smoking medications with the patient. Behavioral support was provided to help manage cravings, withdrawal symptoms, and change routines.

^e^Explored current beliefs about CVD and participants’ responses to learning about the increased CVD risk associated with RA. The important role of lifestyle modifications was discussed, and participants were challenged to determine (using various probing behavioral techniques) and commit to a specific behavior change. Graded goal setting was used as a technique to help them achieve the goal.

^f^The waiting-list participants received an information booklet about the study.

^g^Summary report of non-agreement with the recommendations of the French Society of Rheumatology in 4 RA comorbidities.

^h^Nurses were trained and given a booklet to be used for the systematic identification and assessment of comorbidities (including CVD). Actions taken into account for CVD included smoking cessation (identification of smoking status and calculation of pack-years).

^i^EHR prompts to assess smoking status and 30-day readiness to quit or cut back + advise to quit + electronically connect those willing to receive Quitline support.

#### Risk of bias within studies

S1 Fig shows the traffic-light plot of the risks of bias in the included studies. The randomized controlled trials were rated as having an overall low risk of bias, although concerns about randomization were raised for 2 studies. However, for the nonrandomized trials, all studies were judged to have methodological concerns and 9 were considered to have an overall serious risk of bias.

### Outcomes of interventions targeting patients

#### Smoking cessation

**Fig 2:**
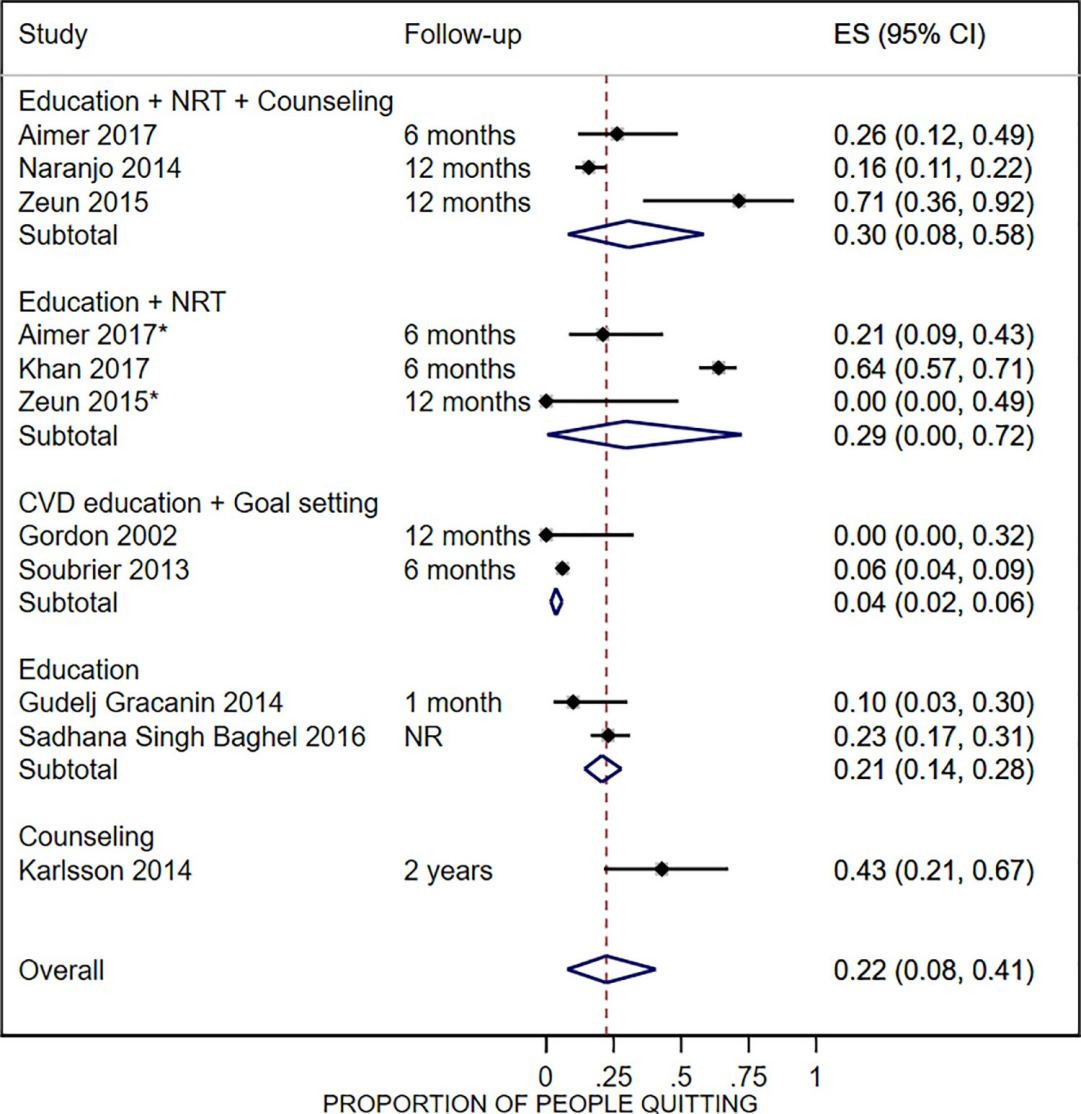
Self-reported smoking cessation rates. *Smoking cessation rates for Aimer 2017 and Zeun 2015 are reported for the intervention and control group in separate rows because both strategies provided smoking cessation services. In the control group in Zeun 2015, patients were referred to their general practitioner for smoking cessation services. ES, effect estimate (proportion of people quitting); CI, confidence interval; NRT, nicotine replacement therapy; CVD, cardiovascular disease. Inconsistency scores for the subgroups (I^2^): Education+NRT+Counseling = 80.2%; Education+NRT = 90.3%; CVD education+Goal setting = 0%; Education = 0%; Counseling = not applicable.

Nine studies reported on this outcome [37,42,44,47,48,50,51,54,56], but only 2 studies defined cessation: 1 as total abstinence in the last 7 days [50] and the other as total abstinence for at least 2 weeks, as verified by a carbon monoxide reading of 0 to 6 parts per million [54]. The remaining studies reported the rates at which participants self-reported quitting smoking without additional details regarding time frame. Rates of smoking cessation varied according to the type of intervention evaluated (Fig 2). The lowest pooled rate was observed in studies evaluating lifestyle education in the context of cardiovascular risk combined with goal setting sessions (4%, 95% CI: 2% to 6%), and the highest rate was reported by a study evaluating counseling sessions for 2 years (43%, 95% CI: 21% to 67%). The pooled rate of smoking cessation from all studies after any intervention (follow-up ranged from 4 weeks to 2 years) was 22% (95% CI: 8% to 41%; I^2^ = 96%; n = 9 studies). Table 3 shows the results of comparative studies evaluating this outcome [37,54,58]. No differences were observed in any of the 3 controlled trials comparing the intervention to a control group.

**Table 3:** Synthesis of results from controlled studies evaluating interventions targeting patients.

Study	Intervention	Control	Outcome	Effect
**Aimer 2017 [37]**	ABC + brief advice and subsidized NRT + additional smoking cessation advice for 3 months	ABC + brief advice + subsidized NRT without the 3 months of sessions	Self-reported smoking cessation rates	RR 1.3 95% CI 0.40 to 4.0
			Reduction in the number of cigarettes	MD 1.3 95% CI −4.4 to 7.0
			Sustained reduction in smoking at 6 months	RR 0.89 95% CI 0.44 to 1.8
**Soubrier 2013 [58]**	Nurse assessed comorbidities (including smoking) + with reports sent to the participant’s physicians	Sham program (video on joint self-assessment plus nurse training on joint self-assessment combined)	Self-reported smoking cessation rates	RR 1.3 95% CI 0.75 to 2.2
**Zeun 2015 [54]**	Patients referred to a general practitioner for smoking cessation services	Patients received 1-year education program with access to the rheumatology clinic stop-smoking services	Self-reported smoking cessation rates	RR 0.76 95% CI 0.44 to 1.3

ABC, ask–brief advice–cessation support; RR, relative risk; CI, confidence interval; MD, mean difference; NRT, nicotine replacement therapy.

#### Percent abstinent

Two studies [47,50] provided the percentage of participants who continued to smoke after the intervention. The pooled rate of abstinent people after the intervention was 21% (95% CI: 11% to 31%). Two studies [53,59] evaluated a cardiovascular risk factors education and found no difference in the number of participants who continued smoking 6–12 months after the intervention (p>0.3). One implementation study with different participants assessed before and after implementation [45] reported similar rates of abstinence in both populations (80%; 95% CI: 75% to 84% vs 78%; 95% CI: 73% to 82%).

#### Reduction in the number of cigarettes

Five studies reported on this outcome. In 1 comparative study, there was no statistically significant difference between groups (Table 3) [37]. Among 3 uncontrolled trials, 1 study reported that only 2 of the 8 participants who were current tobacco users reduced the number of cigarettes smoked a day, but the mean number of cigarettes smoked was not provided [42,43]. At 12 months, Naranjo and colleagues reported a reduction in smoking cigarettes of 30% or greater in 55 of 152 participants and a reduction of 50% in cigarette consumption in 29 of 152 participants [49,50]. Karlson et al. reported a significant reduction in the number of cigarettes smoked before and after the intervention for those participants who continued smoking (MD −9.0, 95% CI: −13.8 to −4.1) [47]. For 1 implementation study with different participants assessed before and after the intervention, the mean (± SD) number of cigarettes smoked per day was similar in the post-implementation and pre-implementation periods (12 ± 7 vs. 14 ± 8, respectively) [45].

#### Knowledge about benefits of smoking cessation

Three studies reported on this outcome. John and colleagues reported mean scores at 6 months on the Heart Disease Fact Questionnaire-Rheumatoid Arthritis (HDFQ-RA), a 13-item questionnaire [46]. The mean (± SD) score in the intervention group, which participated in 2.5-hour education meetings weekly for 8 weeks, was 10.2 ± 2.4, while that of the control group, which was a waiting-list delayed-intervention arm, was 9.1 ± 2.75 (MD: 1.1; 95% CI: 0.13 to 2.1). Tekkatte et al. reported similar scores for participants’ knowledge regarding the importance of smoking on arthritis across 3 groups: current, past, and never-tobacco users (visual analog scale [VAS] mean ± SD: 7.1 ± 2.4, 7.7 ± 1.9, and 7.4 ± 2.2, respectively) [52]. In an implementation study (i.e., one with different participants before and after the intervention) asking participants whether there is a link between RA and smoking, the percentage of adequate responses increased from 5.2% to 25.9%. When participants in this study were asked whether smoking can lessen the effectiveness of RA treatment, the percentage of adequate responses rose from 4.2% to 48.5% [45].

#### Disease measures

Two studies reported data on disease measures. Karlsson et al. compared patients who quit smoking with the intervention versus those who continued smoking at 2 years [47]. The mean pain score of patients who quit smoking improved from 37.5 to 3.0; whereas the mean pain score of patients who continued smoking improved by much less (from 60 to 40; MD: −37.0; 95% CI: −64.7 to −9.3). The global health assessment score decreased from 37.5 to 3.5 in patients who quit smoking, compared to 56.5 to 29.0 in patients who continued smoking (MD: −25.5; 95% CI: −51.9 to 0.91). The mean Health Assessment Questionnaire score improved from 0.57 to 0.0 in patients who quit versus 0.75 to 0.26 in patients who continued smoking (MD: −0.26; 95% CI: −0.84 to 0.32). Similarly, Khan et al. compared participants who quit smoking with participants who continued to smoke. The mean Disease Activity Score-28 for Rheumatoid Arthritis with C-Reactive Protein (DAS28-CRP) was higher in patients who continued smoking than in patients who quit smoking (4.9 vs 2.9, respectively; MD: −2.0; 95% CI: −2.3 to −1.7).

### Outcomes of interventions targeting providers

#### Referrals to smoking cessation services

Four studies provided rates of referral to smoking cessation services before and after implementation in different populations for each period evaluated (S4 Table). In all studies, the pooled rate of referrals increased from pre- to post-implementation (5% to 70% at 3–6 months).

#### Smoking status

Two implementation studies with different participants assessed an intervention for providers before and after implementation [39,41]. The pooled rate of current tobacco users was lower in the post-implementation population at 6 months (15%; 95% CI: 13% to 18%) than in the pre-implementation population (20%; 95% CI: 18% to 21%) (S4 Table).

#### Number of cigarettes smoked

For one implementation study with different participants assessed before and after intervention and unspecified follow-up, the mean (± SD) number of cigarettes smoked per day was similar in the post-implementation period and the pre-implementation period (7 ± 4 vs. 8 ± 5, respectively) [41].

### Heterogeneity

No statistically significant differences were observed in the pooled smoking cessation rates when studies were grouped by design (controlled trials: 11%; 95% CI: 1% to 26%; I^2^ = 76% vs uncontrolled studies: 30%; 95% CI: 11% to 53%; I^2^ = 94%; *p* = 0.12). In addition, no differences in cessation rates were observed between studies with different follow-up periods (≤ 6 months: 23%; 95% CI: 4% to 51%; I^2^ = 98% vs > 6 months: 21%; 95% CI: 3% to 46%; I^2^ = 76%; *p* = 0.98). Finally, the type of intervention used also did not significantly affect smoking cessation rates (with follow-up sessions/contact: 22%; 95% CI: 7% to 41%; I^2^ = 72% vs without follow-up sessions/contact: 23%; 95% CI: 3% to 54%; I^2^ = 98%; *p* = 0.96).

Removing studies that included patients with other rheumatic diseases did not influence the direction or the magnitude of the smoking cessation rate (22%; 95% CI: 2% to 52%; I^2^ = 97.6%). However, removing studies with a high risk of confounding bias resulted in a lower proportion of patients quitting smoking (17%; 95% CI: 6% to 31%; I^2^ = 85%).

### Reporting biases

There was no evidence of small-study effects (Egger test *p* = 0.69) in the funnel plot for the primary outcome assessed (S2 Fig).

### Certainty of evidence

The summary of findings table is shown as S5 Table. The evidence for the main outcome, self-reported cessation rates, was of moderate quality due to limitations in study design and inconsistency of results. Similarly, the evidence for the effects of smoking cessation strategies versus control was of moderate quality due to imprecision and the estimate derived from one small study.

## Discussion

We identified published reports of smoking cessation strategies for patients with RA and synthesized their data on smoking cessation outcomes, referrals to quit services, knowledge about the benefits of cessation, and the benefits of smoking cessation on health outcomes. We found that the success rates of people with RA quitting smoking varied according to strategy used, follow-up duration, and quality of the studies with a pooled cessation rate of 22%. Furthermore, the majority of studies were not randomized trials and several lacked adequate comparator groups, therefore being subject to bias. When high-risk of bias studies were removed, the cessation rates were even lower (17%). This indicates that an optimal and more active strategy for this specific population is yet to be established and tested with a more rigorous methodology.

The most frequent strategy used in the studies included in this review was education with advice to quit smoking delivered in the context of cardiovascular risk. This type of interventions do not conform to current smoking cessation guidelines, in which counseling treatments and medications are recommended [62]. In our review, fewer than half of the studies (41%) reported use of telephone calls, emails, and counseling sessions to provide follow-up support. Included studies reporting on interventions that included counselling and/or NRT achieved greater cessation rates. This is similar to the evidence observed from the general population showing that extended counseling with pharmacotherapy enhances sustained abstinence relative to brief, time-limited approaches. Although for patients with RA, there may need to be some tailoring to cover the specific needs of these patients. Prior studies have explored the specific problems that tobacco interventions need to address in patients with RA [63–66]. Patients have expressed the need for counseling that highlights the relationship between smoking and RA to understand better the short- and long-term complications. They also wanted to learn how to replace smoking for alternatives when smoking was used as a coping mechanism for the frustrations of living with the disease or as a distraction from pain, in particular when most patients with active or severe disease find it difficult to exercise and use it as an alternative distraction. Finally, they prefer alternatives that help them overcome feeling unsupported and isolated from other patients with RA.

One noteworthy finding is that the included controlled trials reported similar cessation rates between groups in 3 different clinical scenarios: (1) comparing education and advice combined with NRT plus counseling versus the same elements without the counseling sessions; (2) comparing a nurse-administered program to evaluate cardiovascular risk factors and other comorbidities combined with sending a report to providers versus a sham program; and (3) education and advice given in the rheumatology clinic versus a general practice of the patient’s choice. The study with the first scenario explored the specific problems that tobacco interventions need to address in patients with RA [37,57]. All participants (intervention and control group) were offered cessation support if they wanted to quit and NRT. They also were counseled to highlight the relationship between smoking and RA to understand better the short- and long-term complications. However, participants in the intervention group were instructed on how to replace smoking for alternatives when smoking was used as a coping mechanism for the frustrations of living with the disease or as a distraction from pain, in particular when most patients with active or severe disease find it difficult to exercise and use it as an alternative distraction. They also received access to a support website to help them overcome feeling unsupported and isolated from other patients with RA. Although the smoking cessation rates in the intervention group were higher compared to the control group, the difference did not reach statistical significance. Both groups achieved smoking cessation rates higher than those rates reported for the general population when no intervention is received [67]. This finding supports previous expert opinion that even a brief general advice intervention not necessarily tailored to specific barriers can increase smoking cessation rates and that more complex or intensive interventions add only small benefits compared to standard care of care. This hypothesis will need to be further tested given that the pooled rate of smoking cessation from all the included studies was lower than the published average cessation rates for minimal interventions in the general population (22% vs 50–60% [68]). Some qualitative studies have explored barriers to and facilitators of smoking cessation in rheumatology clinics. This evidence suggests that psychosocial factors (e.g., patients’ feeling that smoking is ‘the one thing they still have control over’ while dealing with the burden of rheumatic disease), judgmental cessation support from rheumatology staff, lack of investment from providers (e.g., every provider recommending quitting without providing a solution), and lack of cessation education resources are the main barriers to quitting for patients with RA [69]. Patients’ readiness to quit and use of NRT were the most common facilitators of smoking cessation, and visible health effects and the cost of cigarettes were found to be the factors with the most influence on the desire to quit [63,69].

Another important agenda for future research is to find the best choice of outcome measures in this population. Apart from smoking abstinence, the number of cigarettes consumed per day for participants who continued smoking after receiving the intervention and improvement in knowledge about the benefits of smoking cessation after participants received the intervention have also been reported. It is unknown if these outcomes would translate in benefits in disease outcomes. To date only one study has showed improvement in pain, disability, and disease activity scores for patients who quit smoking using structured counseling every 4 weeks over 2 years compared to those who continued to smoke [47]. Future studies focusing on smoking cessation interventions in patients with RA should ensure that disease outcomes are measured given the highly clinical relevance of the measures and considering that many tobacco users with RA report smoking to cope with disease symptoms. To our knowledge, no other reviews have attempted to synthesize the effects of smoking cessation interventions specifically for patients with RA. One Cochrane review considered studies in patients with chronic autoimmune inflammatory joint diseases, but only 2 studies (also included in this review) could be synthesized narratively with no attempt to combine results of the individual studies [70].

Our study has limitations, including a lack of comparative evidence for most strategies reported and large heterogeneity in the populations, definitions of cessation, settings, methodologies used, and points of assessment, which restricted our ability to synthesize the effects. Moreover, most of the meta-analyses performed showed substantial inconsistency scores reflecting substantial clinical, methodological, and statistical heterogeneity. Nonetheless, the trends observed in our results seem to indicate that interventions that include counseling or another type of follow-up method (rather than a 1-time interaction) achieve greater cessation rates. In addition, only limited evidence was reported regarding the effects of smoking cessation on disease-related outcomes, which as evidence by prior qualitative data, RA symptoms are one of the listed reasons for continuing smoking. Furthermore, none of the studies compared cessation rates according to RA treatment received, functional ability, or other medical characteristics that could be helpful to determine the subpopulations that could most benefit from cessation strategies. Another concern was the serious risk of bias observed in the majority of the nonrandomized studies (i.e., 10 out of 18 studies were uncontrolled trials assessing the same participants before and after the intervention and 5 were implementation studies with different participants assessed before and after the intervention). Most such studies did not account for confounding factors (e.g., socioeconomic status, previous smoking cessation attempts, mental health). Moreover, the cessation outcomes were biased given that all studies but one used participant self-reports, rather than biochemical methods, to verify smoking status. Although self-reported smoking cessation is a simple and convenient method, prior studies have shown that participants do not always accurately report their smoking status [71], which can yield inaccurate estimates of the proportion of people quitting. Therefore, the level of certainty of the evidence was considered moderate to very low. This was further exacerbated by the limited information reported in studies published as conference abstracts (10 out of 18). Finally, our results should be carefully interpreted. The cessation rates reported in the studies were substantially heterogeneous which may not only reflect the different population characteristics and cessation strategies used but could also be attributed to different implementation intensities of the interventions evaluated. Adequate research studies with more robust methodology still need to be conducted to identify the effectiveness of smoking cessation programs targeting patients with RA. One promising randomized controlled trial comparing the effect on disease activity of an intensive smoking cessation intervention versus standard of care is still in progress, and its results have not yet been reported [72]. Thus, it is imperative that further well-designed studies be conducted to confirm the results observed in this review.

In conclusion, there was substantial heterogeneity due to differences in patient characteristics, the interventions used, and their implementation structure. Only 3 studies were controlled clinical trials and smoking cessation rates were similar across controlled and uncontrolled trials. However, the rates observed were even lower when removing studies with high risk of bias and considering the likelihood of overestimation due to the self-reported nature of the primary outcome measure, our findings suggest that additional studies evaluating stronger interventions based on current smoking cessation guidelines are needed to facilitate smoking cessation in patients with RA.

## Supporting information

S1 Fig Traffic-light plots showing the risk of bias of individual studies. (DOCX)

S2 Fig Funnel plot for smoking cessation. (DOCX)

S1 Table Medline (Ovid) search strategy. (DOCX)

S2 Table List of excluded studies. (DOCX)

S3 Table Participant characteristics. (DOCX)

S4 Table Synthesis of results of interventions targeting providers. (DOCX)

S5 Table Summary-of-findings table. (DOCX)
