# Supplementary material for: A systematic review with meta-analysis of the effects of smoking cessation strategies in patients with rheumatoid arthritis
Source: PLoS One. 2022 Dec 15;17(12):e0279065. doi: 10.1371/journal.pone.0279065 (PMC9754184; doi:10.1371/journal.pone.0279065)
Supplement: S1 Fig — (DOCX) [file pone.0279065.s001.docx]

**S1 Fig.** **Traffic-light plots showing the risk of bias of individual studies.**


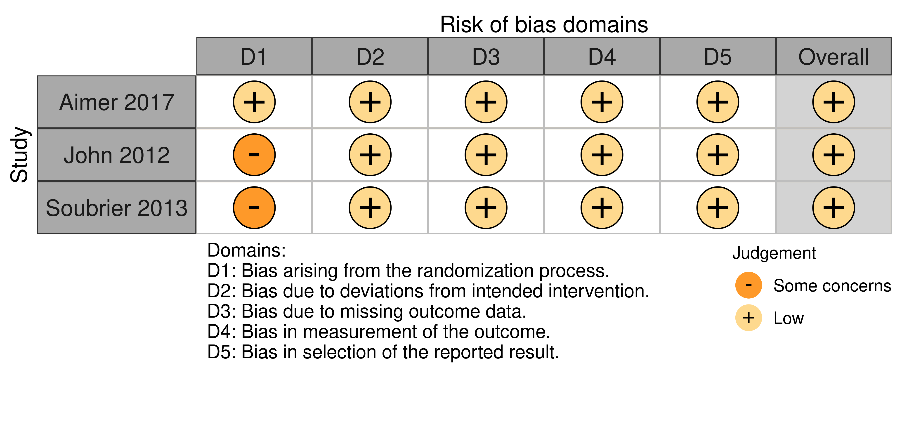


Suppl. Fig 1a. Risk of bias of randomized controlled trials


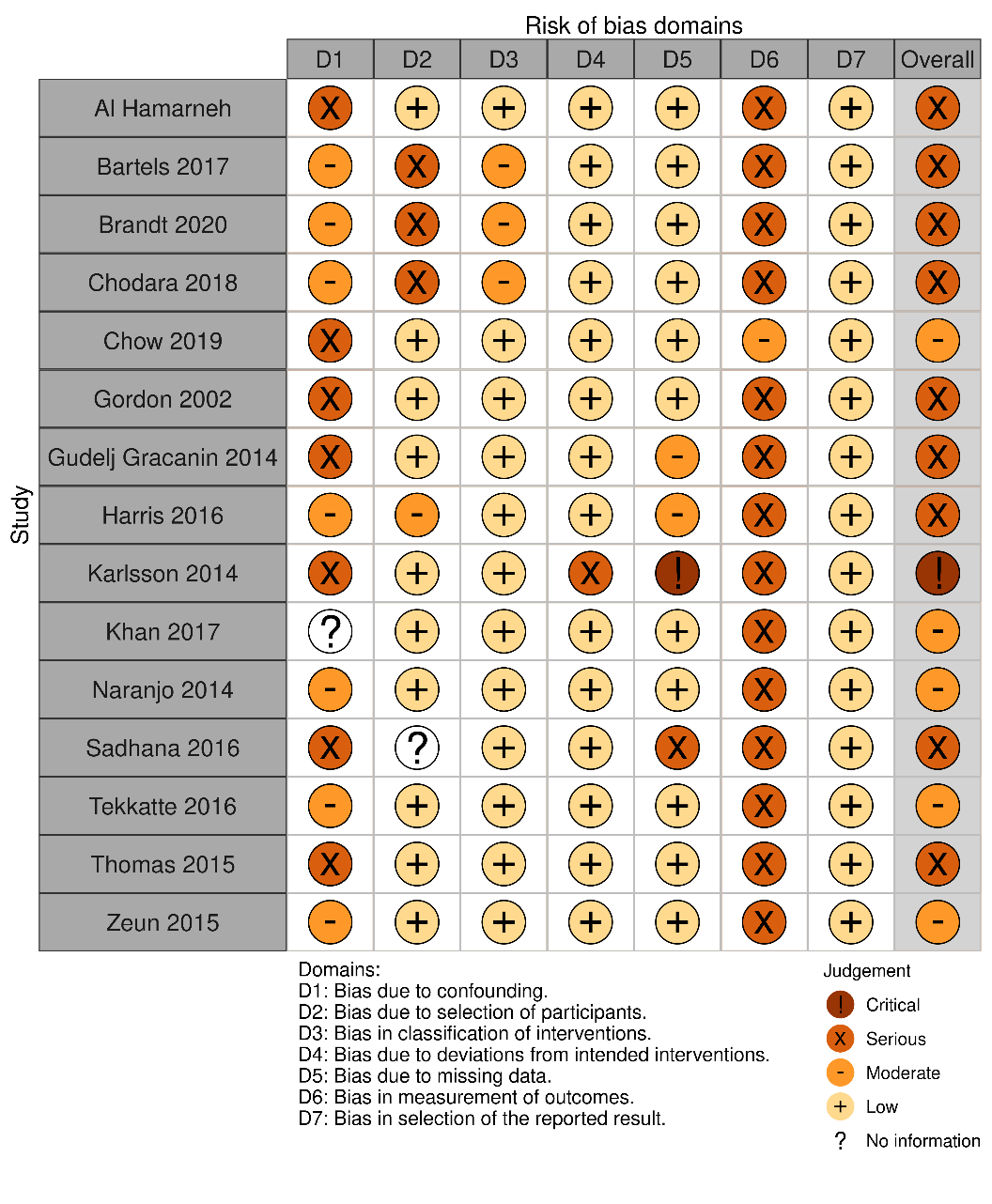


Suppl. Fig 1b. Risk of bias of nonrandomized studies
