# Supplementary material for: A systematic review with meta-analysis of the effects of smoking cessation strategies in patients with rheumatoid arthritis
Source: PLoS One. 2022 Dec 15;17(12):e0279065. doi: 10.1371/journal.pone.0279065 (PMC9754184; doi:10.1371/journal.pone.0279065)
Supplement: S2 Fig — (DOCX) [file pone.0279065.s002.docx]

**S2 Fig.** **Funnel plot for smoking cessation**.

(Egger test *p* = 0.69)

ES, effect estimate (proportion of people quitting; se, standard error.]
