# Supplementary material for: A systematic review with meta-analysis of the effects of smoking cessation strategies in patients with rheumatoid arthritis
Source: PLoS One. 2022 Dec 15;17(12):e0279065. doi: 10.1371/journal.pone.0279065 (PMC9754184; doi:10.1371/journal.pone.0279065)
Supplement: S1 Table — (DOCX) [file pone.0279065.s003.docx]

**S1 Table. Medline (Ovid) search strategy^a^.**

| **Line** | **Terms** |
| --- | --- |
| **1** | Smoking Cessation/ |
| **2** | (Smoking ADJ4 (cessation or quit*)).ti,ab. |
| **3** | OR/1-2 |
| **4** | Arthritis, Rheumatoid/ |
| **5** | ((rheumatoid or reumatoid or revmatoid or rheumatic or reumatic or revmatic or rheumat* or reumat* or revmarthrit*) ADJ3 (arthrit* or artrit* or diseas* or condition* or nodule*)).ti,ab. |
| **6** | OR/4-5 |
| **7** | AND/3,6 |
| **8** | exp animals/ not humans.sh. |
| 9 | 7 NOT 8 |

^a^Date searched March 15, 2022.
