# Supplementary material for: A systematic review with meta-analysis of the effects of smoking cessation strategies in patients with rheumatoid arthritis
Source: PLoS One. 2022 Dec 15;17(12):e0279065. doi: 10.1371/journal.pone.0279065 (PMC9754184; doi:10.1371/journal.pone.0279065)
Supplement: S2 Table — (DOCX) [file pone.0279065.s004.docx]

**S2 Table. List of excluded studies.**

| **#** | **References** |
| --- | --- |
| **1** | Aimer P, Stamp LK, Stebbings S, Cameron V, Kirby S, Croft S, Treharne GJ. Developing a tailored smoking cessation intervention for rheumatoid arthritis patients. *Musculoskeletal Care* 2016; 14(1); 2-14. |
| **2** | Aimer P, Stamp LK, Stebbings S, Cameron V, Kirby S, Treharne GJ. Exploring perceptions of a rheumatoid arthritis-specific smoking cessation programme. *Musculoskeletal Care* 2018; 16(1): 74-81. |
| **3** | Aimer P, Stamp LK, Stebbings S, Cameron V, Kirby S, Treharne G. Exploring perceptions of a rheumatoid arthritis specific smoking cessation programme. *Arthritis Rheumatol.* 2016; 68 (Supplement 10): 1482-1483. |
| **4** | Baer P, Raynauld JP, Dunne S, Remillard MA, Lamb A, Shawi M. Smoking cessation measures in rheumatology practices: results from a self-reflective chart audit. *J Rheumatol.* 2016; 43 (6): 1158. |
| **5** | Gath ME, Stamp LK, Aimer P, Stebbings S, Treharne GJ. Reconceptualizing motivation for smoking cessation among people with rheumatoid arthritis as incentives and facilitators. *Musculoskeletal Care* 2018; 16(1): 139-146. |
| **6** | Bender S, Weinblatt M, Duch D. Measuring Quality Improvement from CME Participants: Results from the RAPID CME Initiative. *Arthritis and Rheumatology* 2020; 72(suppl 10): 3422-3423. |
| **7** | Dougados M, Soubrier M, Perrodeau E, Gossec L, Fayet F, Gilson M, Cerato MH, Pouplin S, Flipo RM, Chabrefy L, Mouterde G, Euller-Ziegler L, Schaeverbeke T, Fautrel B, Saraux A, Chary-Valckenaere I, Chales G, Dernis E, Richette P, Mariette X, Berenbaum F, Sibilia J, Ravaud P. Impact of a nurse-led programme on comorbidity management and impact of a patient self-assessment of disease activity on the management of rheumatoid arthritis: results of a prospective, multicentre, randomised, controlled trial (COMEDRA). *Ann Rheum Dis* 2015; 74(9); 1725-33. |
| **8** | Gath ME, Stamp LK, Aimer P, Stebbings S, Treharne GJ. Reconceptualizing motivation for smoking cessation among people with rheumatoid arthritis as incentives and facilitators. *Musculoskeletal Care* 2018; 16(1): 139-146. |
| **9** | https://clinicaltrials.gov/show/nct02901886 |
| **10** | Kathleen FH, Young-Il K, Meifang C, Rekha R, Maria P, Rajani SS, Thomas KH, William CB. Web-Based Intervention for Transitioning Smokers From Inpatient to Outpatient Care: an RCT. *American journal of preventive medicine* 2016; 51 (4): 620‐629. |
| **11** | Lewis D, Capell HA, McNeil CJ, Smith WE, Brown DH. Cigarette smoking and the clinical outcome of gold therapy in rheumatoid arthritis. *J Rheumatol* 1984; 11(1): 111-2. |
| **12** | Liu X, Leatherwood C, Tedeschi SK, Barbhaiya M, Speyer C, Lu B, Costenbader K, Karlson E, Sparks JA. Impact and timing of smoking cessation on reducing risk for seropositive rheumatoid arthritis among women. *Arthritis and Rheumatology* 2018; 70 (Supplement 9): 2131-2132. |
| **13** | McKay ND, Hamilton J, Saravanan V, Kelly C. Audit of smoking and attitude to smoking cessation in rheumatoid arthritis. *Rheumatology* 2007; 46: I85-I86. |
| **14** | Miller J, Knight L, Altham A, Gunasekera W, Sharma P, Dahiya S. Smoking cessation strategies in RA: what would it take to make them work? *Rheumatology (United Kingdom)* 2018; 57 (Supplement 3): iii163. |
| **15** | Panyard D, Ramly E, Gilmore-Bykovskyi A, Lauver D, Adsit R, Maxcy C, Bartels CM. Developing a staff-driven electronic smoking cessation referral program in rheumatology clinics. *Arthritis Rheumatol* 2016; 68 (Supplement 10): 550-552. |
| **16** | Roelsgaard IK, Esbensen BA, Ostergaard M, Rollefstad S, Semb AG, Christensen R, Thomsen T. Smoking cessation intervention for reducing disease activity in chronic autoimmune inflammatory joint diseases. *Cochrane Database of Systematic Reviews* 2018 (2) (no pagination) CD012958. |
| **17** | Roelsgaard IK, Thomsen T, Ostergaard M, Christensen R, Hetland ML, Jacobsen S, Andersen L, Tonnesen H, Rollefstad S, Semb AG, Esbensen BA. The effect of an intensive smoking cessation intervention on disease activity in patients with rheumatoid arthritis: study protocol for a randomised controlled trial. *Trials* 2017; 18(1): 570. |
| **18** | Roelsgaard IK, Thomsen T, ostergaard M, Semb AG, Andersen L, Esbensen BA. Evaluation of patients' experiences of an intensive smoking cessation intervention for people with rheumatoid arthritis. *Ann Rheum Dis.* 2019; 78 (Supplement 2): 185. |
| **19** | Roelsgaard IK, Ikdahl E, Rollefstad S, Wibetoe G, Esbensen BA, Kitas GD, Van Riel P, Gabriel S, Kvien TK, Douglas K, Jonsson SW, Dahlqvist SR, Karpouzas G, Dessein P, Tsang L, El-Gabalawy H, Hitchon C, Pascual VD, Contreras-Yanez I, Sfikakis P, Gonzalez-Gay MA, Crowson CS, Semb AG. Smoking cessation in patients with RA is associated with reduced CVD event rates and improved lipid profiles and predicts lower RA disease activity. Annals of the Rheumatic Diseases 2019; 78 (Supplement 2): 1121-1122. |
| **20** | Sparks JA, Chang SC, Nguyen UDT, Barbhaiya M, Tedeschi SK, Lu B, Costenbader KH, Zhang Y, Choi HK, Karlson EW. Smoking behavior changes in the early rheumatoid arthritis period and risk of mortality during thirty-six years of prospective followup. *Arthritis Care Res (Hoboken)* 2018; 70(1): 19-29. |
| **21** | Stamp LK, Gath M, Aimer P, Stebbings S, Treharne G. Promoting smoking cessation among rheumatoid arthritis patients: what motivations and barriers are reported after being offered a smoking cessation intervention? Conference: American College of Rheumatology/Association of Rheumatology Health Professionals Annual Scientific Meeting, ACR/ARHP. *Arthritis Rheumatol.* 2015; 67 Suppl. 10. |
| **22** | Wattiaux A, Bettendorf B, Block L, Gilmore-Bykovskyi A, Ramly E, Piper ME, Rosenthal A, Sadusky J, Cox E, Chewning B, Bartels CM. Patient perspectives on smoking cessation and interventions in rheumatology clinics. *Arthritis Care Res (Hoboken).* 2020; 72 (3): 369-377. |
| **23** | Wattiaux A, Block L, Gilmore-Bykovskyi A, Ramly E, Sadusky J, Piper M, Bettendorf B, Rosenthal A, Bartels CM. Supporting smoking cessation in RA and SLE: identifying patient-centered outcomes. Conference: American College of Rheumatology/Association of Rheumatology Health Professionals Annual Scientific Meeting, ACR/ARHP. *Arthritis Rheumatol.* 2017; 69 Supplement 10. |
