# Supplementary material for: A systematic review with meta-analysis of the effects of smoking cessation strategies in patients with rheumatoid arthritis
Source: PLoS One. 2022 Dec 15;17(12):e0279065. doi: 10.1371/journal.pone.0279065 (PMC9754184; doi:10.1371/journal.pone.0279065)
Supplement: S3 Table — (DOCX) [file pone.0279065.s005.docx]

**S3 Table. Participant characteristics.**

| **Study** | **Setting** | **Age, mean (SD), years** | **Female sex, No. (%)** | **Ethnicity, No. (%)** | **Socio-demographic information** | **Current smokers, No. (%)**^b^ | **Pack-years, mean (SD)** |
| --- | --- | --- | --- | --- | --- | --- | --- |
| ***Randomized controlled trials*** | | | | | | | |
| **Aimer 2017 [37, 57]** | Academic | 56.5 (11.8) | 21 (55) | New Zealand European: 34 (89) | Socioeconomic deprivation score: 5.5 ± 2.7 | All current smokers | 37.8 (22.9)  16.6 (8.2) cig/day |
| **John 2013 [46]** | Community | I = 62.19, C = 60.81 | I = 37 (71), C = 43 (74) | Caucasian: 107 (97) | NR | Current smokers I = 8 (15), C = 11 (19) | NR |
| **Soubrier 2013 [56, 58]** | Secondary/tertiary care rheumatology departments | 58 (11) | 766 (79) | NR | NR | Current smokers I = 66 (13.8), C = 91(19.8) | NR |
| ***Uncontrolled trials (same participants assessed at baseline and follow ups)*** | | | | | | | |
| **Al Hamarneh 2021 [59]** | Community | 64 (14.8) | 60 (61) | Caucasian: 85 (86) | NR | Current smokers 11 (110 | NR |
| **Gordon 2001, 2002 [42, 43]** | Community | 52 | 20 (91) | NR | NR | Current smokers 8 (36) | NR |
| **Gracanin 2014 [44]** | Community | 60.2 (11.99) | 82 (85) | All from continental Croatia | NR | Current smokers 20 (20.8) | NR |
| **Karlsson 2014 [47]** | Academic | NR | NR | NR | NR | All current smokers | 10 cig/day |
| **Khan 2017 [48]** | Community | 56 (11.9) | 137 (76) | NR | NR | All current smokers | NR |
| **Naranjo 2013, 2014 [49, 50]** | Academic | 50 | 98 (64) | NR | NR | All current smokers (>1 cig/day) | 29 (17) |
| **Sadhana Singh Baghel 2016, 2017 [51, 55]** | Academic | NR | 108 (51) | All Indian | 19 (9.0%) illiterate, 79 (37.4%) basic education, 64 (30.3%) graduate, 49 (23.2%) postgraduate. | Current smokers 74 (35) | NR |
| **Tekkatte 2016 [52]** | NR | NR | 74 (74) | NR | 10 unemployed | Current smokers 22 (22) | NR |
| **Thomas 2015 [53]** | Community | 65 | 17 (85) | NR | NR | Current smokers 6 (30) | NR |
| **Zeun 2015 [54]** | Community | 52 | 139 (69) | Caucasian 103 (51%) | NR | Current smokers 36 (18) | 7.2 cig/day |
| ***Implementation studies (different participants assessed before and after intervention)*** | | | | | | | |
| **Bartels 2017 [38, 60]** | Academic | NR | NR | NR | NR | All current smokers | NR |
| **Brandt 2020 [39, 61]** | Community | NR | NR | Black 83% | 40% uninsured, 52% Medicare/Medicaid | All current smokers | NR |
| **Chodara 2018 [40]** | Community | NR | NR | NR | NR | All current smokers | NR |
| **Chow 2019 [41]** | Community | NR | NR | NR | NR | Current smokers B = 16 (30), A = 11 (11) | 2004-2010 = 8 (5) cig/day and 2010-2016 = 7(4) cig/day |
| **Harris 2016 [45]** | Community | 61 | 238 (70) | NR | NR | Current smokers B = 62 (20), A = 75 (22) | NR |

A, after the intervention; B, before the intervention; C, control group; cig/day, cigarettes per day; I, intervention group; NR, not reported; RA, rheumatoid arthritis.

^a^Numbers reflect the total number of patients included in the study (i.e., smokers [active, passive, current, past] or nonsmokers), independently if they received the intervention or not.

^b^Some studies provided data for previous smokers or nonsmokers as control groups not receiving the intervention. Other studies included an intervention that targeted multiple cardiovascular risk factors, and for this study only the smokers were used.
