# Supplementary material for: A systematic review with meta-analysis of the effects of smoking cessation strategies in patients with rheumatoid arthritis
Source: PLoS One. 2022 Dec 15;17(12):e0279065. doi: 10.1371/journal.pone.0279065 (PMC9754184; doi:10.1371/journal.pone.0279065)
Supplement: S4 Table — (DOCX) [file pone.0279065.s006.docx]

**S4 Table. Synthesis of results of interventions targeting providers.**

| **Referrals to Quit lines for smoking cessation** | | | | | | | |
| --- | --- | --- | --- | --- | --- | --- | --- |
| **Study** | **Outcome measured at** | **Pre-implementation** | | | **Post-implementation** | | |
|  |  | **No. of patients referred/Total smokers** | **% (95% CI)** | | **# patients referred/Total smokers** | | **% (95% CI)** |
| **Bartels 2017 [38, 60]** | 3 months | - | 0.6^a^ | | 93/122 | | 76 (68-83) |
| **Brandt 2020 [39, 61]** | 6 months | - | - | | 19/30 | | 63 (46-78) |
| **Chodara 2018 [40]** | NR | 3/100 | 3 (1-8) | | 40/129 | | 31 (24-39) |
| **Chow 2019 [41]** | NR | 4/16 | 25 (10-49) | | 11/11 | | 100 (74-100) |
| *Pooled rate* | | *5 (0-16)* | | *70 (38-94)* | | | |
|  | |  | |  | | | |
| **Smoking status** | | | | | | | |
| **Study** | **Outcome measured at** | **Pre-implementation**^b^ | | | **Post-implementation**^b^ | | |
|  |  | **Current smokers/Total** | **% (95% CI)** | | **Current smokers/Total** | | **% (95% CI)** |
| **Brandt 2020 [39, 61]** | 6 months | *535/2674* | *20 (19-22)* | | *123/780* | | *16 (13-18)* |
| **Chow 2019 [41]** | NR | 16/53 | 30 (20-44) | | 11/100 | | 11 (6-19) |
| *Pooled rate* | | *20 (18-21)* | | | *15 (13-18)* | | |
|  | |  | | |  | | |
| **Number of cigarettes smoked** | | | | | | | |
| **Study** | **Outcome measured at** | **Pre-implementation**^b^ | | | **Post-implementation**^b^ | | |
|  |  | **Mean** | **SD** | | **Mean** | **SD** | |
| **Chow 2019 [41]** | NR | 7 | 4 | | 8 | 5 | |

CI, confidence interval; NR, not reported.

^a^Historical data reported in abstract.

^b^The pre- and post-implementation participants were different subjects.
