# Supplementary material for: A systematic review with meta-analysis of the effects of smoking cessation strategies in patients with rheumatoid arthritis
Source: PLoS One. 2022 Dec 15;17(12):e0279065. doi: 10.1371/journal.pone.0279065 (PMC9754184; doi:10.1371/journal.pone.0279065)
Supplement: S5 Table — (DOCX) [file pone.0279065.s007.docx]

**S5 Table. Summary-of-findings table.**

| **Outcomes** | **No. of participants (studies)**  **Follow-up** | **Quality of the evidence (GRADE)** | **Estimate (95% CI)** |
| --- | --- | --- | --- |
| **Self-reported smoking cessation**  **(ABC intervention + session vs ABC intervention only)** | 38  1 RCT  6 months | ⊕⊕⊕⊖  MODERATE due to imprecision and the estimate derived from 1 small study | RR 1.3, 95% CI 0.40 to 4.0 |
| **Self-reported smoking cessation**  **(Rheumatologist vs GP office)** | 11  1 CT  12 months | ⊕⊕⊖⊖  LOW due to risk of bias, imprecision, and the estimate derived from 1 small study | RR 0.76, 95% CI 0.44 to 1.3 |
| **Self-reported smoking cessation rates** | 1,031  8 (2 RCT, 2 pilot trials, 5 Cohorts)  1 to 12 months | ⊕⊕⊕⊖ MODERATE  due to risk of bias and inconsistency of results | Proportion 22%, 95% CI 8% to 41% |
| **Number of current smokers (after implementation)** | 1,220  3 (3 implementation studies, different population for pre and post assessment)  6 months | ⊕⊕⊕⊖ MODERATE  due to risk of bias and inconsistency of results | Proportion 17% (12% to 22%) |
| **Number of cigarettes smoked (ABC intervention + session vs ABC intervention only)** | 38  1 RCT  6 months | ⊕⊕⊕⊖  MODERATE due to imprecision and the estimate derived from 1 small study | MD 1.3, 95% CI -4.4 to 7.0 |
| **Number of cigarettes smoked (after implementation)** | 175  2 BA  3-12 months | ⊕⊕⊖⊖  LOW due to risk of bias, imprecision, and inconsistency | Weighted mean 9.0 (-22.3 to 40.6) |
| **Knowledge about benefits of smoking cessation** | 110  1 RCT  6 months | ⊕⊖⊖⊖  VERY LOW due to risk of bias, imprecision, indirectness, and the estimate derived from 1 small study | MD 1.1, 95% CI 0.13 to 2.1 |
| **Referrals to Quit lines for smoking cessation (after implementation)** | 1,921  4 BA  3-6 months | ⊕⊕⊖⊖  LOW due to risk of bias, imprecision, and inconsistency | Proportion 70% (38% to 94%) |

ABC, Ask–Brief advice–Cessation support; BA, [please define]; CI, confidence interval; CT, controlled trial; GP, general practitioner; MD, mean difference; RCT, randomized controlled trial; RR, relative risk
